# Antifungal Resistance Regarding *Malassezia pachydermatis*: Where Are We Now?

**DOI:** 10.3390/jof6020093

**Published:** 2020-06-25

**Authors:** Andrea Peano, Elizabeth Johnson, Elisa Chiavassa, Paolo Tizzani, Jacques Guillot, Mario Pasquetti

**Affiliations:** 1 Dipartimento di Scienze Veterinarie, Università di Torino, Largo Paolo Braccini 2, Grugliasco, 10095 Torino, Italy; elisac_85@hotmail.it (E.C.); paolo.tizzani@unito.it (P.T.); mario.pasquetti@unito.it (M.P.); 2 Mycology Reference Laboratory, PHE South West Laboratory, Southmead Hospital, Bristol BS10 5NB, UK; elizabeth.johnson@PHE.gov.uk; 3 EA 7380 Dynamic, Ecole nationale vétérinaire d’Alfort, UPEC, USC Anses, 94704 Maisons-Alfort, France; jacques.guillot@vet-alfort.fr

**Keywords:** *Malassezia pachydermatis*, dog, antifungal resistance, MIC, in vitro susceptibility testing

## Abstract

*Malassezia pachydermatis* is a yeast inhabiting the skin and ear canals in healthy dogs. In the presence of various predisposing conditions it can cause otitis and dermatitis, which are treated with multiple antifungal agents, mainly azole derivatives. This manuscript aims to review the available evidence regarding the occurrence of resistance phenomena in this organism. Various findings support the capacity of *M. pachydermatis* for developing resistance. These include some reports of treatment failure in dogs, the reduced antifungal activity found against yeast isolates sampled from dogs with exposure to antifungal drugs and strains exposed to antifungal agents in vitro, and the description of resistance mechanisms. At the same time, the data reviewed may suggest that the development of resistance is a rare eventuality in canine practice. For example, only three publications describe confirmed cases of treatment failure due to antifungal resistance, and most claims of resistance made by past studies are based on interpretive breakpoints that lack sound support from the clinical perspective. However, it is possible that resistant cases are underreported in literature, perhaps due to the difficulty of obtaining a laboratory confirmation given that a standard procedure for susceptibility testing of *M. pachydermatis* is still unavailable. These considerations highlight the need for maintaining surveillance for the possible emergence of clinically relevant resistance, hopefully through a shared strategy put in place by the scientific community.

## 1. Introduction

The genus *Malassezia* includes different yeast species, some of which are a cause of dermatological diseases in humans and animals [1]. *Malassezia pachydermatis* has been traditionally considered the lone species within the genus to be not strictly lipid-dependent [1]. However, recent studies [2,3] have revealed that the gene encoding the fatty acid synthase is missing in all *Malassezia* species, indicating that the whole genus is lipid-dependent. The idea that *M. pachydermatis* was lipophilic but not lipid-dependent was based on the observation that most isolates can grow in Sabouraud-dextrose agar (SDA) without added lipids [1]. This phenomenon has been explained by the discovery that the commercial peptone used to prepare SDA contains small amounts of palmitic acid and other fatty acids [2].

*M. pachydermatis* is a commensal organism commonly found on the skin, in ear canals, and on the mucosal surfaces of healthy dogs. Favourable growth conditions in the local environment allow the excessive multiplication of this organism, which may then function as an opportunistic secondary pathogen [4,5,6]. Dogs may present with *Malassezia* otitis (MO), dermatitis (MD) (either localised or generalised), or both [6].

Though *M. pachydermatis* predominates in dogs, other species, such as *M. furfur*, have been occasionally reported in cases of dermatitis or otitis [4].

*M. pachydermatis* has also been associated with dermatitis and otitis in cats. Still, these clinical entities are considered to be rare compared with dogs, in which MO is extremely common, and MD is moderately common [6]. Though *M. pachydermatis* is of great interest principally in veterinary practice, it has also been reported as a sporadic agent of bloodstream infections in humans, immune-compromised patients, and neonatal units [7,8].

MD and MO in dogs are generally treated by systemic and topical therapy using several antifungal agents with different mechanisms of action, in addition to various antiseptics. The most commonly used antifungals are azole derivatives, though other agents belonging to various chemical classes are also used [9,10,11,12,13,14,15,16,17]. Antifungal treatments are generally successful in controlling yeast overgrowth, but failure or rapid recurrence occasionally occur. The primary reason for failure is usually thought to be the lack of identifying and resolving primary causes and predisposing factors [9,11,18,19], while the role played by the possible phenomena of antifungal resistance is still poorly defined.

Unlike what has been done for bacterial and other fungal infections, the scientific community has not yet adopted a shared strategy to address the problem of antifungal resistance in *M. pachydermatis*. In particular, a standard procedure for in vitro susceptibility testing—which is essential in order to obtain reproducible and clinically significant measurements of antifungal activity—has not been developed yet [20]. Based on different in vitro tests, some studies have reported high resistance rates for various agents [21,22,23,24]. According to some authors, the appearance of resistant strains would be a concern [25], while a low azole susceptibility of the yeast would be a well-documented phenomenon [26]. Instead, other studies have reported a substantial efficacy against yeastby the antifungal agents of more common use in dogs [27,28,29].

This manuscript aims to review the available evidence regarding the occurrence of resistance phenomena in *M. pachydermatis*.

## 2. Background

### 2.1. Malassezia otitis/Dermatitis in Dogs

#### 2.1.1. Pathogenesis and Clinical Signs

The overgrowth of yeast is encouraged in presence of a cutaneous microenvironment modified by a primary inflammatory process, particularly in the course of diseases that cause increased moisture, increased sebum production, altered surface lipids, the disruption of the stratum corneum barrier function, or aberrant immune responses [4].

Processes involved in colonisation and infection include the adherence of the yeast to stratum corneum cells, the secretion of hydrolases, and the innate and adaptive immune responses of the host [5]. Underlying conditions leading to MD include hypersensitivity diseases (atopic dermatitis, adverse cutaneous food reactions, flea bite hypersensitivity, and contact allergy), keratinisation disorders, ectoparasite infections, bacterial pyoderma, endocrine diseases (hyperadrenocorticism, hypothyroidism, diabetes mellitus), and autoimmune diseases [6,30]. Cases of MD without apparent underlying causes have been also reported [31]. Genetic predisposition appears to be important in certain breeds, especially West Highland white terriers, Basset hounds, dachshunds, cocker spaniels, Shih Tzus, and English setters [30].

**Figure 1:**
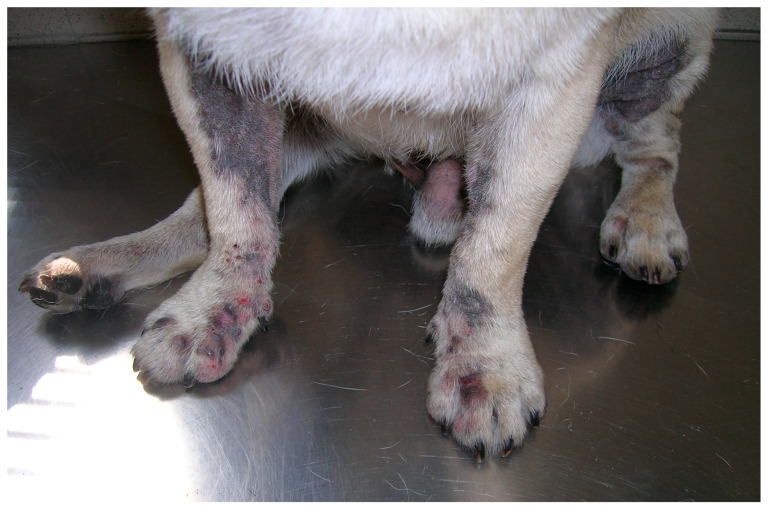
Hyperpigmented inflammatory lesions in a dog with *Malassezia* dermatitis secondary to a *Demodex* mites infestation.

MD is typically intensely pruritic, with erythema as the primary lesion produced. Secondary lesions are common and include excoriations, seborrheic plaques, lichenification, hyperpigmentation, maceration, and intertrigo. The inflamed skin can either be dry and flaky or greasy (seborrhea oleosa). In generalised cases, a rancid odour is commonly reported [6] (Figure 1).

*M. pachydermatis* also plays an important role in cases of ceruminous otitis externa, in which it is often highly pro-inflammatory [6]. Frequently, ear and skin localisations occur together [6].

**Figure 2:**
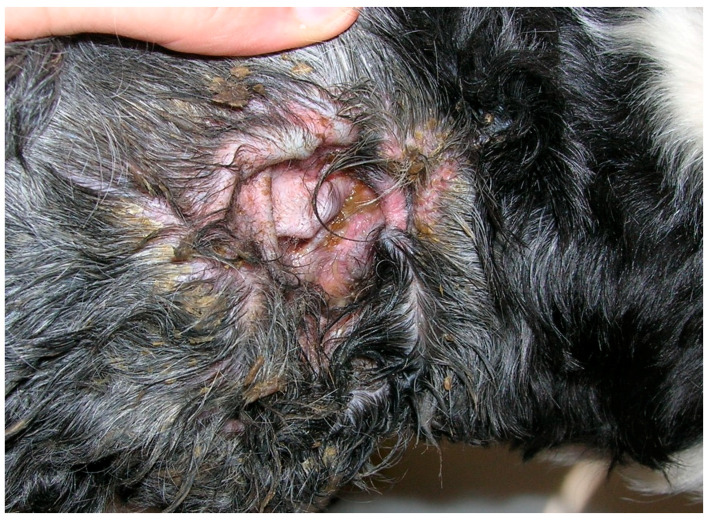
*Malassezia* otitis in a dog.

Underlying conditions that predispose a dog to the development of MO include anatomical anomalies or changes that create ear canal stenosis, increased cerumen secretion or retention, moisture, and the inhibition of air circulation. Under these conditions, primary inflammatory diseases of the ear canal, which can occur as part of atopic dermatitis or adverse food reactions, is a common cause of MO. Other causes of ear inflammation include foreign bodies, parasites (especially the ear mite *Otodectes cynotis*), keratinisation disorders, and autoimmune diseases [5,6,9,19]. Dogs with MO show erythematous external ear canals accompanied by a waxy to seborrhoeic yellow or brownish discharge [5] (Figure 2).

A hypersensitivity response to the yeast itself is likely to occur in many allergic dogs [5,6].

**Figure 3:**
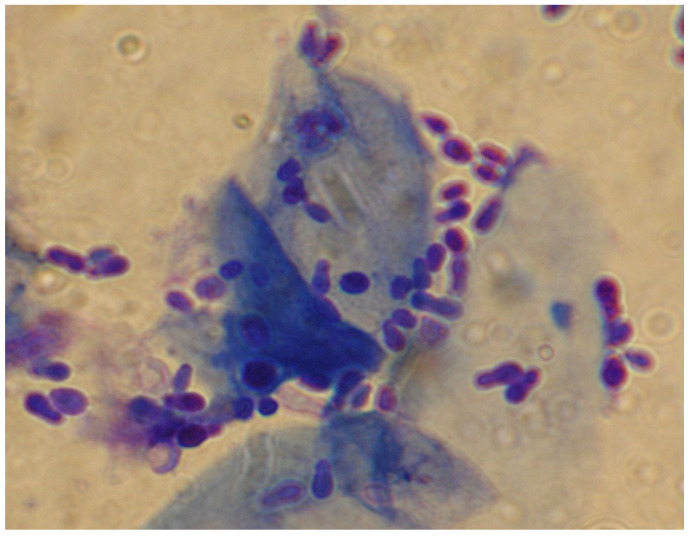
Skin cytology from a dog with *Malassezia* dermatitis. Numerous typical peanut-shape budding yeasts (1000-fold magnification, Hemacolor^®^ rapid staining kit).

Diagnosis is based on detecting the yeast on cytology from compatible skin lesions and ear discharge (Figure 3) and observing a clinical and mycological response to empirically selected therapy [5,6,18].

#### 2.1.2. Antifungal Treatments

Current options for the treatment of MD and MO include, in addition to various antiseptics such as selenium sulphide and chlorhexidine, systemic and topical therapy with several antifungal agents [4,9,10,11,12,13,14,15,16,17,18,32]. The most commonly used antifungals for dogs belong to various chemical classes with different mechanisms of action (Table 1).

Polyene macrolides, natural products of the *Streptomyces* species, are a class of poorly absorbed, large macrocyclic polyketides that interact with membrane sterols [12]. Within this class, nystatin (NYS) is used in the topical treatment of canine MO [18].

Several antifungal agents belong to the group of azole-containing compounds, which function by interacting with sterol-14α-demethylase, involved in the biosynthesis of ergosterol [12]. These agents represent the most common choice for treating MO and MD in dogs. Some of the azole derivatives—for example, miconazole (MCZ), clotrimazole (CTZ), and econazole (ECZ)—are available for topical application only. Ketoconazole (KTZ) is used both orally and topically, while the newer triazoles—fluconazole (FCZ) and itraconazole (ITZ)—are administered orally [9,11,33]. Posaconazole (PSZ) is a triazole that has been recently introduced in human medicine, where it has progressively acquired importance for the treatment of invasive fungal infections. An aural PSZ-based formulation for dogs has also been recently developed.

Allylamines form another group of antifungal agents which perturb fungal sterol synthesis by inhibiting squalene epoxidase [12]. Terbinafine (TER) belongs to this class and has been reported to be effective with oral treatment for *Malassezia* dermatitis [15].

The final agent of interest for the control of *Malassezia* overgrowth in the dog is thiabendazole (TBZ), which belongs to the group of benzimidazoles, the activity of which is based on the inhibition of microtubule assembly in yeast cells [12]. TBZ is used topically and is included in an aural formulation for dogs.

Many of the agents mentioned above are marketed for veterinary use, particularly in products for topical application, such as shampoos, dermatological solutions, and aural formulations. Generally, aural formulations are products which also contain glucocorticoids and antibiotics. Veterinary medicines containing these agents are less widely available for systemic use. Therefore, formulations for human medicine are often used off-label [12,33].

Since yeast (and bacterial) infections of the skin and ear canals are thought to be secondary problems in most cases, it is universally agreed that it is of utmost importance also to identify and resolve the primary underlying condition [9,11,18,19]. On the other hand, it is essential to remember that in some cases, especially in predisposed breeds, there is no identifiable underlying cause and the dog’s skin disease resolves completely with antifungal therapy [30].

Another polyene macrolide, amphotericin B (AMB), though very active against *M. pachydermatis* [34] is not employed to treat MD and MO in dogs for reasons of toxicity, the difficulty of usage (an intravenous administration is necessary), and costs.

### 2.2. Drug Resistance and In Vitro Tests

Drug resistance is a well-known problem regarding many infective microorganisms, above all bacteria, but also some species of yeasts of medical importance, such as *Candida* spp. and, to a lesser extent, the *Cryptococcus neoformans*-*Cryptococcus gattii* complex [35,36,37,38,39,40,41,42].

The mechanisms of antifungal resistance are related to the intrinsic or acquired characteristics of the fungal pathogen that interfere with the antifungal mechanism of the respective drug, or that lower target drug levels [38]. The phenomenon of “drug-resistance” is well characterised in the above-mentioned yeasts thanks to the existence of standardised procedures for in vitro antifungal susceptibility testing (AFST), developed through years of collaborative work [35,37]. Establishing a set of standardised criteria for in vitro testing is essential, since different testing variables are known to have an impact on in vitro determinations [35,36,37]. Thus, by modifying test conditions, microbial isolates could appear to be either susceptible or resistant [35].

#### 2.2.1. Antifungal Resistance: Definitions

Antifungal resistance can be defined as being microbiological or clinical [38]. Microbiological resistance is said to occur when an antimicrobial agent inhibits the growth of the pathogen only at concentrations higher than the range observed for wild-type (WT) strains (the term WT refers to an isolate without acquired resistance mechanisms). Breakpoints defined in this way are known as epidemiological cut-off values (ECOFF or ECV). Clinical resistance is determined by the situation in which the infecting organism is inhibited only by a concentration of an antimicrobial agent that is associated with a higher likelihood of therapeutic failure. In other words, the pathogen is only inhibited by an antimicrobial concentration that is higher than could be safely achieved with normal dosing. Breakpoints defined in this way are known as clinical breakpoints (CBP) [38].

#### 2.2.2. Mechanisms of Resistance

The definitive confirmation of the possibility of drug resistance comes from the description of the underlying molecular mechanisms. For *Candida* spp., these include the following in regard to azole drugs: (1) reduced affinity of the target (lanosterol demethylase) for the azole; (2) an energy-dependent efflux mechanism that causes the decreased intracellular accumulation of azoles; (3) up-regulation of target enzyme, with the antifungal agent being consequently overwhelmed; and (4) the development of bypass pathways, through which ergosterol is replaced by its precursor 14α-methylfecosterol, with the latter product leading to still-functional membranes [39]. Regarding *C. neoformans*, resistance has been demonstrated to occur from an upregulation or modification of the target enzyme, reduced access of the drug to the target, or combinations of these mechanisms [40,43].

#### 2.2.3. Testing Methods for Antifungal Resistance

The recognised methods for the AFST of yeasts are those by the Clinical and Laboratory Standards Institute (CLSI, formerly the National Committee for Clinical Laboratory Standards (NCCLS)) [44,45] and the European Committee on Antibiotic Susceptibility Testing (EUCAST) [46]. The methods, which are intended for testing *Candida* spp. (CLSI and EUCAST) and *Cryptococcus neoformans* (CLSI), rely on the measurement of growth inhibition during exposure to a range of doubling drug concentrations diluted in a liquid medium. The results are thus expressed as the minimum concentration of the drug able to inhibit fungal growth (minimum inhibitory concentration [MIC]) [36].

A standard antifungal disk diffusion susceptibility testing method is now also available for *Candida* spp. (CLSI document M44-A2). In this case, the results are expressed as inhibition diameters [36,47]. Other possibilities for the susceptibility testing of yeasts include some commercial kits that have potential advantages in terms of ease of use, flexibility, and the rapidity of results [36]. These kits, developed and intended for testing *Candida* spp., are based on a broth dilution format more or less similar to that of the reference methods or other formats, such as the *E*-test^®^. This latter is a plastic test strip impregnated with a continuous concentration gradient of an antifungal agent. This way, an MIC can be obtained through an agar diffusion test by considering where the border of the inhibition zone intercepts the graded MIC scale on the strip.

It is almost universally accepted that the conditions employed for *Candida* are not suitable for testing *M. pachydermatis* due to the different physiologic features. For example, the medium recommended in the CLSI assay - RPMI (Roswell Park Memorial Institute) broth - is lipid-free, while *M. pachydermatis* needs a lipid supplementation to reach an adequately vigorous growth [29,48,49,50]. The lipid sources more frequently employed are tween, glycerol, olive oil, oleic acid, ox bile, and cow’s milk fat [20].

Moreover, *M. pachydermatis* has a slower growth rate compared to that of *Candida* species and tends to form clusters [29]. Therefore, different adjustments have been adopted in time, as discussed deeply in a recent review [20]. Though the experience of various research groups has allowed identifying the technical parameters adapted for the AFST of *M. pachydermatis* [20], guidelines dedicated explicitly to this yeast are not available yet.

#### 2.2.4. The In Vitro Antifungal Susceptibility of *M. pachydermatis*

In most studies, the MICs appear to be distributed over a range of values around the modal MIC (e.g., ITZ 0.01–1 µg/mL [51]; FCZ 0.01–4 µg/mL [51]; MCZ 0.03-16 µg/mL [29]). Such a distribution is generally shown in a WT organism population (to be exact, the typical MIC distribution for WT organisms covers three to five twofold dilution steps surrounding the modal MIC [52,53]). Reports of substantially similar MICs of an agent for all strains are less common [27].

**Figure 4:**
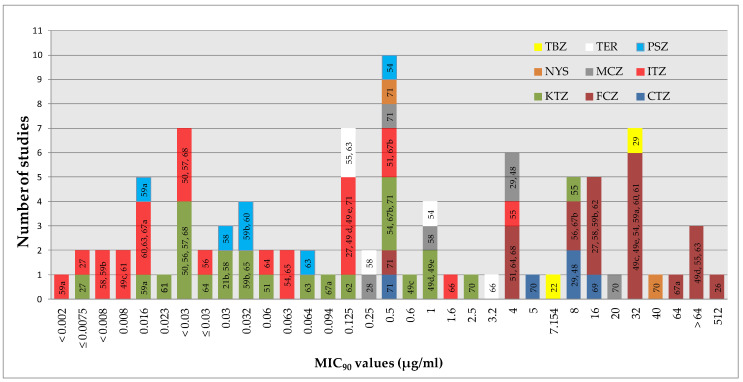
MIC_90_ values obtained for different antifungal agents against *M. pachydermatis* and the number of studies that reported each value (MIC_90_ is the MIC required to inhibit the growth of at least 90% of the isolates tested). References are inside the bars. a = MICs obtained with an E-test; b = MICs obtained with the BMD method; c, d, e = MICs obtained in Sabouraud Broth + Tween, Urea Christensen Broth + Tween, and Dixon broth, respectively. Due to space limitations, the values refer to the overall isolates tested in each study; eventual results regarding specific subgroups of strains within a study (e.g., coming from animals with/without lesions) are not included. KTZ = ketoconazole; MCZ = miconazole; CTZ = clotrimazole; ECZ = econazole; ITZ = itraconazole; FCZ = fluconazole; PSZ = posaconazole; NYS = nystatin; TER = terbinafine; TBZ = thiabendazole.

A graphical summary of the activity reported for different antifungal agents in some studies [22,26,27,28,29,48,49,50,51,54,55,56,57,58,59,60,61,62,63,64,65,66,67,68,69,70,71] (Figure 4) allows appreciating the variability in results obtained thus far.

Support for the idea that variability is largely determined by the use of different test conditions comes from the MICs reported for a reference strain (CBS 1879, considered the type strain of *M. pachydermatis*) (KTZ: <0.03 µg/mL [50], 0.25–0.5 µg/mL [72], 1.25 µg/mL [70]; ITZ: ≤0.03 µg/mL [73], 1.6 µg/mL [66]; TER: ≤0.03 µg/mL [73], 3.2 µg/mL [66]; NYS: 0.63 µg/mL [70], 5 µg/mL [74]) and the results obtained in some studies after the application of two different tests or different test conditions (e.g., ITZ MIC_90_ by a broth microdilution technique: 0.5 µg/mL, by *E*-test^®^: 0.016 µg/mL [67]; ITZ MIC_90_ in Christensen urea broth + Tween 40/80: 0.125 µg/mL, in Sabouraud Broth + Tween 80: <0.008 µg/mL [49]).

Regardless of this variability, it is possible to draw a ranking with regard to the absolute potency against *M. pachydermatys* by the different agents tested. ITZ and PSZ were the most active agents (MIC_90_ for most studies ≤0.5 µg/mL), followed by KTZ (MIC_90_ ≤1 µg/mL), whereas FCZ had higher absolute values (>4 µg/mL in most cases). With regard to the other agents, the MIC_90_ went from 0.25 to 20 µg/mL (MCZ), 0.125 to 3.2 µg/mL (TER), 0.5 to 40 µg/mL (NYS), 0.5 to 16 µg/mL (CTZ), and 7 to 32 µg/mL (TBZ).

#### 2.2.5. Criteria for Interpreting In Vitro Results

The MIC values (like any other measurement) obtained in vitro are indicators of the relative susceptibility of fungi to the agents under consideration. Still, these values alone are insufficient to predict the clinical outcome in vivo. Instead, these measurements are more practically linked with the therapeutic outcome when integrated with clinical information and pharmacokinetic (PK) data to provide interpretive clinical breakpoints (CBPs) [37,38,75]. CBPs are thus specific MIC values (or diameters of inhibition zones) that enable fungi to be assigned to the clinical categories “susceptible (S)”, “intermediate (I)” (also known as “susceptible-dose dependent (S-DD)”, and “resistant (R)”. Intermediate susceptibility implies that an infection caused by the isolate can be treated effectively at body sites where the antifungal drug is physiologically concentrated, or when a high dosage of drug can be used [37,38,75].

It is now clear that, in addition to the intrinsic drug susceptibility, the final therapeutic outcome in the course of infections by *Candida*, as for any infectious disease, depends on several other factors. These include factors regarding drugs (e.g., impaired drug absorption, accelerated drug metabolism, and poor penetration into the site of infection), general host factors (e.g., inflammatory response, hypersensitivity reactions, phagocyte function, and underlying diseases), and factors pertaining to pathogen activity (e.g., toxin production and other virulence factors). For these reasons, both CLSI and EUCAST have established CBPs, taking into account the MIC distributions, PK data (such as the maximum concentration of drug in the serum) and pharmacodynamic (PD) parameters, resistance mechanisms, and clinical outcomes, as these all relate to MIC values [36,37,38]. Importantly, as many factors are involved in determining the clinical outcome of a fungal infection, in vitro susceptibility does not always predict a successful therapeutic outcome, while in vitro resistance often, but not always, predicts therapeutic failure [38,75,76]. This is clearly illustrated by the so-called “90-60 rule”, which states that infections due to strains of *Candida* spp. deemed as susceptible respond to appropriate therapy in ≈90% of cases, whereas infections due to resistant strains respond in ≈60% of cases [38].

Tentative breakpoints were originally set by the CLSI for FCZ and ITZ against *Candida* spp. [77]. They have since been replaced by species-specific breakpoints in the light of greater clinical and susceptibility testing experience [38,44]. Breakpoints are now also available for other antifungal agents, such as voriconazole, flucytosine, and echinocandins [36,38]. For FCZ, caspofungin, and voriconazole, interpretive breakpoints have also been established by the CLSI (again for *Candida* spp.) with the disk diffusion format. In this case, the breakpoints are expressed in mm (i.e., the size of the inhibition zones) [36].

There are no recommendations by the CLSI/EUCAST for interpreting the results for other drugs (e.g., MCZ, CTZ, KTZ, TER, etc.). However, tentative breakpoints can be found for some of them in the instruction manuals provided by manufacturers of commercially available tests—either agar disk/tablet diffusion testing or MIC-based kits [78]. These indications are generally intended for *Candida* infections.

Different authors have recently utilised data from global antifungal surveillance MIC databases to ascertain the WT MIC distribution for various agents against different *Candida* species and, as such, have established the so-called epidemiological cut-off values (ECVs) [52,53,79]. In this construct, the ECVs represent the upper limit of the wild-type MIC distribution and set a cut-off for detecting the emergence of reduced susceptibility or acquired resistance. This information has been utilised for establishing species-specific breakpoints that have been recently defined to account for the diverse susceptibility profiles shown by different *Candida* species [52,53,79]. ECVs provide a sensitive means for identifying isolates that are less likely to respond to therapy when limited clinical data preclude the development of CBPs [52].

As regards the *C. neoformans-C. gattii* species complex, for which CBPs are not available, ECVs have been defined for AMB, flucytosine, and various azole agents (FCZ, ITZ, PSZ, and voriconazole) [40,80]. For this fungal group, epidemiological studies point out that ECVs should be species-specific or even molecular type-specific [40].

Officially recognised interpretive criteria of in vitro results—both CBPs and ECVs—are not available for *M. pachydermatis*.

#### 2.2.6. Antifungal Susceptibility Profile and Possible Resistance Phenomena Regarding Other Malassezia Species

*M. globosa*, *M. restricta*, and *M. sympodialis* are the predominant Malassezia species on human skin. They are involved in various dermatological diseases, such as atopic dermatitis, dandruff/seborrheic dermatitis, and pityriasis versicolor. Another species, *M. furfur*, has also been reported as an agent of bloodstream infections in immune-compromised patients and neonatal units [8,34].

Evidence suggests that the susceptibility to AMB, azoles, and TER varies according to species. *M. sympodialis* (together with *M. pachydermatis*) is the most susceptible, while *M. furfur* and *M. globosa* are the least susceptible species [34]. ITZ and KTZ are the most active agents against all Malassezia species, while FCZ and voriconazole are the least active [34].

The correlation of antifungal susceptibility with clinical outcome has been rarely reported and deserves further investigation. As regards FCZ, high MIC values correlated well with poor clinical responses in patients with fungemia by *M. furfur* [34]. On the contrary, high AMB MIC values were detected in *M. furfur* strains coming from patients with a positive clinical outcome. This discrepancy may be due to the unsuitability of the methods employed to test the in vitro susceptibility. Alternatively, the positive outcomes of patients might be due to the synergic effect of additional drugs [34].

As regards the mechanisms of resistance in these Malassezia species, Iatta et al. [26] suggested that the azole resistance of *M. furfur* may depend on drug efflux pumps. In another study [81], KTZ-resistant *M. restricta* strains from patients with dandruff were shown to possess tandem multiplications of the ERG11 gene encoding a lanosterol 14α-demethylase, which is the direct target enzyme of azole antifungal drugs. In the same study, the authors observed an increased drug efflux in a resistant isolate, suggesting that this also influences resistance in *M. restricta*.

## 3. Method Employed for the Review

Studies for this review were included based on a literature search via the Pubmed and Scopus databases using the following key terms: *Malassezia pachydermatis*, MIC, resistance, and susceptibility testing. Additional pertinent citations were then identified in the bibliography of papers that had been selected. To identify possible contributions from “grey literature” (doctoral dissertations, conference papers, etc.), a request was sent to the Vetderm Listserv (vetderm@lists.ncsu.edu), a forum open to veterinarians all over the world, who have an interest in animal skin diseases.

Publications were excluded if they focused on drugs that are not used in dogs affected with *Malassezia* dermatitis/otitis (i.e., AMB, voriconazole, caspofungin, flucytosine), on isolates from human bloodstream infections, on the activity of antiseptics or natural compounds, or on the response of dogs to antifungal therapies without the in vitro measurement of the activity of the drugs employed. Only English-language original studies were included in this review.

The lack of a standard method for the AFST of *M. pachydermatis* prevents a possible attribution of resistance for an isolate with an MIC higher than MICs obtained by other authors (or inhibition zone smaller), on the basis that this isolate may appear susceptible under different test conditions. On the other hand, we must consider the possibility that resistance was present in isolates with higher MICs or smaller inhibition zones compared to other strains tested with the same method—i.e., within each study. The results obtained, for example, by Brito et al. (2007) [27]—an MIC of ITZ ≤ 0.0075 µg/mL for all the isolates tested—are not particularly suggestive of the presence of resistance because the MICs were very low, but above all because no strain stood out due to a higher MIC. The same consideration applies to a study that, on the opposite side, obtained much higher absolute MICs of ITZ—1.6 µg/mL [66]—but, again, for all the isolates tested.

Taking into account this reasoning, we individuated the studies whose results may support the existence of resistance phenomena in *M. pachydermatis* (e.g., cases of treatment failure, claims of resistance based on various interpretive breakpoints, MICs higher for strains sampled after antifungal therapies, the possibility of the in vitro induction of resistance). The selected studies are summarised in Document S1 (Supplemental Material) and commented on in the following sections. Document S1 is an excel file structured to allow a quick consultation of the main results reported in the studies analysed, with details on the methodology employed for susceptibility tests. From the main page, the reader can access each study’s description by clicking on the authors’ names. The publications are reported in the order of data.

## 4. Reports of Treatment Failure due to Resistance

Three publications—two recent case reports [82,83] and a prospective study [84]—provided strong evidence in support of the presence of clinically relevant azole resistance in isolates coming from a total of seven dogs. These studies were based on the same stringent approach—namely, (1) isolates were obtained from dogs where resistance was suspected on a clinical basis; (2) to overcome the problem of the lack of reference methods for AFST, at least two methods were employed; (3) the MICs (or inhibition zones) for the isolates from the suspected cases were compared with the MICs obtained for “control cases” (dogs responding to therapy, dogs never subjected to antifungal treatments, and reference strains of the yeast). For one of the case reports [83], resistance was confirmed at the molecular level (see Section 12).

The prospective study [84] was conducted in a veterinary clinic in Australia. The criteria for trial admission were the presence of MO, MD, or both; *Malassezia* yeasts identified on cytology; and the failure to respond to typically clinically effective empirically selected antifungal therapies. The correlation of a clinical lack of response with in vitro results was noted for five out of eight chosen cases (Table 2). To justify the three cases with the clinical suspicion of resistance not confirmed by in vitro tests, authors recalled the consideration mentioned above that a failure of response might occur because of several factors. For two dogs, they suspected that there had been problems with the topical drug administration. Host factors were claimed for the remaining case (the dog was affected by a severe keratinisation defect) [84].

As regards the two other publications, the first concerned a dog in Japan [83] (additional information about this case was provided in a subsequent article [85]). The clinical case was not described in detail. At the same time, more considerable attention was given to the study of possible mechanisms of resistance in the strain of yeast isolated from the dog (see Section 12).

The other publication [82] included an exhaustive description of the case, which was followed for an extended period (Table 2 and Document S1). This case, found in Italy, was “idiopathic” as possible underlying systemic or dermatological problems were carefully and repeatedly ruled out by extensive clinical and laboratory investigations. Therefore, the only way of controlling the clinical signs was a continuing course of azoles (oral ITZ and topical MCZ), which ultimately resulted in the development of resistance. Interestingly, the treatment failure occurred after a very prolonged successful treatment (2.5 years), which may indicate that resistance to *M. pachydermatis* develops very slowly. The same finding supports the fact that resistance to the isolates of the yeast was an acquired characteristic rather than an intrinsic feature. The Australian study too may indicate that resistance to *M. pachydermatis* is an acquired slow-developing phenomenon. Indeed, the authors reported that “resistant” cases in the 12 months previous to sampling had a mean of 4.4 courses of antifungal medications, while the dogs with “sensitive” isolates had only a mean of 0.8 courses [84]. As regards the Japanese case, information about previous antifungal treatments was not clear. In a table, the column “treatment history” reported “Shampooing with 2% of MCZ and 2% chlorhexidine”, but it was not specified whether this treatment had been active before the clinical failure occurred [85]).

An in vitro result common to these publications [82,83,84] is that, for many isolates coming from the “resistant” cases, an MIC (as well as an inhibition zone) of different antifungal agents was not obtained (Table 2). This result may indicate a complete lack of—or at least a highly reduced—efficacy by the antifungal agents under testing. This finding supports the correlation between in vitro results and treatment failure as regards topically employed principles. For topical antifungals, resistance based on increased MIC values (measured in microgram/mL) would be indeed poorly significant from a clinical perspective, since topical medications may have a 1000-fold higher concentration (milligrams/mL).

Other findings regarding the Italian case [82] are worth citing. A different degree of in vitro susceptibility was noted for one of the isolates (no MIC of MCZ and ITZ detected, Document S1). This suggests that the skin of a given dog may be colonised by strains of *M. pachydermatis* with different antifungal susceptibility profiles (a similar result was also obtained for a dog in the Australian study). Even azole agents that had not been used in the dog (PSZ, FCZ, KTZ) showed a reduced activity in vitro. This may confirm that the phenomenon of the cross-resistance of *M. pachydermatis* to different azoles demonstrated during in vitro experiments [51] (see Section 10) also occurs in vivo. Finally, the MICs for the isolates sampled at a later date (11 months after the first sampling) were consistently higher than those for the control isolates. This may indicate that the resistance developed in the yeast population harboured by a given dog is stable over time.

## 5. Claims of Resistance

Some studies [21,22,23,24,49,50,51,58,59,61,62,67,70,86,87] evaluating the antifungal susceptibility of *M. pachydermatis* reported variable percentages of isolates resistant to various agents (Document S1) based on different interpretive breakpoints. However, in none of these studies were the in vitro values correlated with the lack of response to therapy.

### 5.1. Interpretation Based on MIC_50_/MIC_90_ Values

In some cases [21,22,58,59,61,62,67], breakpoints were selected taking into account the MIC_50_/MIC_90_ values (MIC_50_ and MIC_90_ are the MICs required to inhibit the growth of at least 50% and 90% of the isolates tested, respectively. MIC_50_ corresponds to the median). Therefore, strains were regarded as S if the MIC of the strain was ≤ MIC_50_; I/SDD if the MIC of the strain was between the MIC_50_ and MIC_90_; and R if the MIC of the strain was > MIC_90_. None of the studies provided a convincing explanation for the use of this interpretive criterion. At best, some authors [22,61,67] justified their choice by stating that this rule had been established in two past works. However, one of these works [88] is not available for consultation, given that it is an abstract presented at a congress held in 1994 and not present in any public database. The other one [89] is focused on different *Candida* species and *C. neoformans* and does not include any proposal for an S/I/R categorisation based on the MIC_50_/MIC_90_ values. One of the final sentences of the manuscript simply states that “a drug concentration equal to its MIC_90_ was considered the susceptibility breakpoint”, and that this cut-off was adopted arbitrarily.

Although the MIC_50_ and MIC_90_ values serve to describe and summarise the distribution of MICs for a population of organisms, they hardly work as CBPs because they do not mirror the concentrations that the tested drugs can reach in infected tissues. For example, considering the serum levels of ITZ reached in dogs [90] and the concentration in the stratum corneum and sebum [10], most of the MIC_90_ values proposed as breakpoints—0.008 µg/mL [58,61], 0.002 µg/mL [59], 0.016 µg/mL [67], 0.125 µg/mL [62]—and even the highest MICs reported are low enough that they are likely to be exceeded during treatment. Some doubts may remain for some MICs obtained by Nascente et al. (2003) [67]—namely, 2–4 µg/mL. The same reasoning applies to KTZ in the case of oral therapy [91], despite the less favourable skin/plasma ratio [92,93]. Additionally, for KTZ an exception may be the MICs obtained for a few isolates (1–8 µg/mL) [67].

These PK-based considerations apply a fortiori to KTZ when used topically and to drugs used topically only—namely, MCZ, PSZ, and TBZ. These agents are principally used to treat *Malassezia* otitis in dogs (though MCZ is also available in formulations for dermatological use in many countries). An organism that is reported to be resistant based on the finding of increased MIC may not be resistant to the high concentration of antifungal that can be safely delivered locally on the skin and within the ear canal itself [18]. Although topical use does not necessarily imply that all of the administered agent is subsequently bio-available to the tissues (given that the drug uptake into the skin depends on several factors, such as the formulation of the commercial product, the molecular mass of the agent, and its inherent ability to penetrate and accumulate in the sebum and the stratum corneum [94,95]), the concentrations of topically administered antimicrobials often reach levels that largely exceed those achieved in the circulation [18]. Previous studies have shown that the clinical response to topically applied antibiotics does not correlate with antimicrobial susceptibility results [96].

Regarding TER, the breakpoint value indicated by Cafarchia et al. (2012) [58] (0.25 µg/mL) approximates the levels that the agent has been shown to reach in the canine stratum corneum during oral treatment [97]. This breakpoint may be incidentally more significant from a PK perspective, but no isolate had an MIC higher than this value [58].

The results obtained for FCZ are more challenging to evaluate. The MICs much higher than those found for the other agents indicate a minor absolute potency of this agent. However, it does not follow that this drug will fail, provided those concentrations can be exceeded at the site of infection. In this regard, data obtained in humans are encouraging [98,99], with levels in the stratum corneum potentially exceeding most of the MICs obtained for FCZ against *M. pachydermatis* in studies employing the MIC_50_/MIC_90_ rule. Though it has been hypothesised that FCZ also accumulates in the stratum corneum of dogs [14]—with peak plasma levels of approximately 10 µg/mL following a 10 mg/kg dose [100]—we cannot know for sure whether this accumulation is of the same entity as that shown in humans. Regardless of these considerations, the attribution of resistance appears justified for some isolates with MICs ≥ 256 µg/mL [59,61,67], which are very unlikely to be exceeded in tissues.

Another problem is that the MIC_90_ varies case by case depending on the distribution studied, while, for a given agent, the breakpoint should always be the same. Moreover, the percentage of resistant isolates would be fixed at no more than 10%, given that the MIC_90_ encompasses at least 90% of the strains tested by definition. In this regard, how the percentage of resistant isolates in some studies [21,22,67] employing the MIC_50_/MIC_90_ criterion could be over 10% is unclear (Document S1).

If the use of the MIC_90_ value as the resistance breakpoint has little clinical relevance, employing MIC_50_ as a separate susceptibility breakpoint appears to be even more dubious. In this way, an intermediate category (I or S-DD) is generated, which seems to be unrealistic when applied to the interpretation of MICs obtained for *M. pachydermatis*. The I and S-DD categories correspond to a well-defined situation during *Candida* infections, in which clinical efficacy may be obtained when higher than usual dosages of a drug can be used and the maximal possible blood/tissue levels achieved [38]. Such categories remain meaningless if the supporting clinical studies have not been conducted and, from a PK perspective, are irrelevant for *Malassezia*. The lack of relevance is particularly evident in the case of topical agents, as discussed above, and in the case of the very low, almost incidental, MIC_50_ and MIC_90_ values reported in one of the studies analysed [61].

In theory, the MIC_90_ value could be re-evaluated for use as an epidemiological cut-off value (ECV) to detect the emergence of strains of with reduced susceptibility to a specific agent, following the lead for *Candida* spp. [79]. Indeed, we previously mentioned that the ECV corresponds to the upper limit of the MIC ranges obtained for a WT population, and in general it encompasses at least 95% of isolates in the WT distribution [52].

### 5.2. Breakpoints Developed for Candida spp

Cafarchia et al. (2012) [49], in their study aimed at verifying the impact of the culture medium on the results of susceptibility tests for *M. pachydermatis*, stated that they had employed the tentative breakpoints established for different azole compounds on *Candida* spp. by Rex et al. (1997) [77] (ITZ: S ≤ 0.125 µg/mL, S-DD = 0.25–0.5 µg/mL, R ≥ 1 µg/mL; FCZ: S ≤ 8 µg/mL, S-DD = 16–32 µg/mL, R ≥ 64 µg/mL). These values were proposed after a highly articulated study conducted by different researchers of the NCCLS and are based on PK, epidemiological, and clinical considerations regarding *Candida* infections in human patients. Therefore, their clinical significance may be dubious when applied to a different fungus/host combination (*M. pachydermatis*/dog). This consideration is reinforced by the notion that even for the same pathogen/host combination, the same breakpoint may not work if applied to a different clinical form. For example, for ITZ and *Candida*, the breakpoints are based entirely on experience with mucosal infections, while for echinocandins and voriconazole, the data are substantially based on non-neutropenic patients with candidemia. Consequently, it has been suggested that their clinical relevance in other settings should be considered with caution [44].

Moreover, we have already expressed reservations about the use of an S-DD category for *M. pachydermatis*. Regarding KTZ, the situation is quite obscure, as breakpoints for this agent are not present in the study by Rex et al. (1997) [77] or the CLSI guidelines [44,45]. It is thus unclear from where the values employed by Cafarchia et al. (2012) [49] (S < 8 µg/mL, R > 16 µg/mL) were taken. Regardless of these considerations, some of the MICs obtained may be indicative of resistance (e.g., >16 µg/mL for one and two isolates against ITZ and KTZ, respectively). However, the MICs were significantly lower using a different medium (Sabouraud dextrose broth with Tween), demonstrating once again that, by modifying test conditions, yeast isolates can be made to appear susceptible or resistant.

For their study, Jesus et al. (2011) [51] used the breakpoints developed for *Candida* cited at the beginning of this section. With this approach, no resistance was found for FCZ (MICs for 30 field isolates 0.01–4 µg/mL), and only 80% of the strains were considered to be susceptible to ITZ. However, the strains deemed as being S-DD or R had MICs from 0.125 to 1 µg/mL and, as discussed above, such a level of ITZ may be reached in the epidermal layer during treatment for MD in a dog. For KTZ, the situation appears unclear because no information is provided about the breakpoints used, with the authors simply reporting that all the strains were susceptible to this agent.

### 5.3. Resistance if MIC is Significantly Higher

This interpretive approach was employed in a study [50] describing “the first report” of a particular isolate of *M. pachydermatis* resistant to KTZ and ITZ. The MICs of both drugs were significantly higher in that isolate (1–2 µg/mL for KTZ; 2–8 µg/mL for ITZ) than in the other 29 strains tested (0.03 µg/mL for both ITZ and KTZ). Such a finding may justify the attribution of resistance by these authors. However, the final sentence of the paper stated that the strain deemed resistant came from a dog with seborrheic dermatitis, for which “the response to KTZ therapy was not different from that of other cases with susceptible yeasts”. We presume this means that all of the dogs were cured, which makes the finding of a significantly higher MIC clinically irrelevant in this case, at least as far as KTZ therapy is considered.

### 5.4. MIC not Reached

Uchida et al. (1990) [70] reported isolates that were considered resistant to two drugs used topically—MCZ (one strain) and NYS (three strains)—as the MICs for these strains were higher than the highest concentration tested (80 µg/mL). All of the other isolates were considered susceptible, provided that a MIC was established. Accordingly, no resistance was claimed for CTZ and KTZ.

We believe that this interpretation has a sound rationale for drugs used in a topical application. As discussed above, topical formulations include antifungal agents at concentrations of mg/mL, thus several-fold higher than MICs whose order of magnitude is µg/mL. It, therefore, seems sensible that only a complete lack of in vitro efficacy (i.e., the inability to determine a finite MIC or complete lack of a zone of inhibition) could predict clinical resistance. This consideration finds support in the results reported for clinically resistant cases [82,83,84,85] (Section 4), where an MIC of topically employed azoles was not found for isolates of *Malassezia* coming from dogs poorly responding to therapy.

On the other hand, by attributing importance to the absence of an MIC, the authors [70] did not note that their results could suggest resistance to oral therapy with KTZ. Indeed, the high MIC of this agent obtained for some strains (2.5–10 µg/mL) might not be reached in the epidermal layer of a dog after the oral intake of KTZ tablets due to the PK properties of this drug.

### 5.5. Breakpoints Provided by the Producers of Disks

Rougier et al. (2005) [87], using an agar disk diffusion method, reported that approximately 4% of isolates were resistant to CTZ, MCZ, and NYS, in addition to variable percentages of isolates deemed as intermediate (9.4% for CTZ, 7.5% for MCZ) based on the “zone interpretive standard breakpoints for veterinary pathogens” provided by the producers of the disks. We think that such an approach can be regarded as too generic, given that a breakpoint can be considered to be relevant if it is defined separately for each combination of fungal infection (with its clinical features, underlying conditions, intrinsic drug susceptibility, etc.) and drug (with its PK, dosages, method of administration, etc.). The same consideration applies to the study by Lyskova et al. [86], who found resistance to FCZ for approximately 4% of the isolates tested. In this case, the authors referred to the “zone diameter interpretive standards for microscopic fungi”, again defined by the suppliers of the disks. Other concerns about the study by Rougier et al. (2005) [87] regard the use of an intermediate category for topical agents; that the authors failed to specify whether the isolates that they classified as R had smaller inhibition zones or no inhibition at all, which is important because, for an agent used in topical preparations, the complete absence of an inhibition zone may provide more robust support for possible clinical resistance; and that the sum of the percentages of S, I, and R strains presented in the paper does not add up to 100% (e.g., for MCZ S = 77.4%, I = 7.5%, and R = 3.8%).

### 5.6. Undefined Breakpoints

Bernardo et al. (1998) [23], who even reported “multi-resistance” profiles (e.g., towards CTZ, MCZ, NYS, 5-fluorocytosine, and AMB), did not specify the cut-off values used to categorise the isolates they tested, rendering their findings poorly supported.

### 5.7. Unclear Results

Lorenzini et al. (1995) [24] reported that TBZ and other agents not examined in the present review exhibited no activity against the strains tested. We can presume that they demonstrated a lack of inhibition zones, but this could have been more clearly stated. The procedure outlined in the study lacks detail, and the presentation of the results is obscure. Moreover, only five isolates were tested. Accordingly, the conclusions drawn about the ineffectiveness of TBZ may be questionable. Support for this is provided by a recent study [29] that reported the MIC values of TBZ for all the strains tested based on a robust experimental approach (i.e., a modification of the CLSI procedure).

## 6. MICs Higher for Isolates from Dogs with Lesions than Isolates from Healthy Dogs

Three independent studies [58,71,101] reported that the MICs of various antifungal agents were significantly higher for isolates from animals with otitis/dermatitis than isolates from healthy animals. In two cases [58,101], it was hypothesised that increased MICs were due to the exposure of isolates from symptomatic animals to antifungal drugs, which may support the idea that resistance can develop during treatment. Although information on the clinical history and eventual treatments (agents employed, dosages, length of therapy, etc.) of the sampled animals was not available, Watanabe et al. (2014) [101] explained that “due to the fact that *Malassezia* is considered to be an exacerbating factor of canine atopic dermatitis (AD), we expect that many of the cases of canine AD were treated with either oral or topical azoles”. It is also worth noting that the study by Watanabe et al. (2014) [101] reported MICs that appear significant from a PK perspective (up to 21 µg/mL for both KTZ and ITZ). However, in the other two studies the differences between MICs for isolates from dogs with/without lesions, although deemed significant for some agents, appear to be clinically irrelevant—e.g., the mean MIC of MCZ: isolates from healthy dogs 0.061 µg/mL, isolates from dogs with otitis 0.124 µg/mL [71].

## 7. MICs Higher for Isolates from Dogs with Chronic Otitis

Chiavassa et al. (2012) [48] tested the antifungal susceptibility of isolates of *M. pachydermatis* obtained from cases of chronic otitis that had been previously treated with various topical ear products containing MCZ and CTZ. These isolates were associated with significantly higher MIC values for both the agents compared to isolates from dogs with acute forms of the disorder that had never received antifungal treatments. Although the authors themselves observed that increased MICs (CTZ 2–16 µg/mL; MCZ 1–8 µg/mL) are unlikely to have any clinical relevance due to the topical use of the agents being tested, these findings may indicate the possibility of resistance developing during treatment.

## 8. MICs Higher in Genetic Subgroups

One study [59] compared the antifungal susceptibility of three major genotypes of *M. pachydermatis* (A, B, C) distinguished through multilocus sequencing using the chitin synthase-2 (chs-2) gene, the first internal transcribed spacer (ITS-1), and the large subunit (LSU) of nuclear rDNA. The susceptibility of different genotypes was also compared within two clinical subgroups: isolates from animals with/without skin lesions. Considering all of the isolates, significantly higher mean MICs were found for genotype B isolates towards FCZ. Additionally, the MIC_50_ values of KTZ and PSZ were higher for genotype B isolates from dogs with skin lesions than those from animals without lesions. In contrast, the mean MIC for KTZ was significantly higher in genotype A isolates from dogs without lesions than those from dogs with skin lesions (Document S1). Based on these results, the authors stated that their data support the hypothesis, formulated previously by themselves [58] and other authors [102], that “host skin may be responsible for the selection of specific genetic populations of *Malassezia* yeasts having an indirect effect on their drug susceptibility”. They also affirmed that their results “suggest a direct correlation between specific genotypes and in vitro drug susceptibility”. Actually, the differences, even those deemed significant, appear minimal in most of the tested agents (e.g., MIC_50_ for ITZ < 0.008 µg/mL for all genotypes; mean MIC for PSZ 0.018, 0.02, and 0.016 µg/mL for genotypes A, B, and C, respectively; MIC_50_ for KTZ genotype B, isolates from dogs with lesions 0.032 µg/mL, isolates from dogs without lesions 0.016 µg/mL; mean MIC of FCZ considering all isolates 10, 15.7, and 10.6 µg/mL for genotypes A, B, and C, respectively). Moreover, the reported MICs appear to be clinically irrelevant from a PK perspective.

Other authors reported results that appear to contrast with the hypothesis of a correlation between genotype and antifungal susceptibility. Álvarez-Pérez et al. (2016) [54], using a fluorophore-enhanced PCR fingerprinting method, found very high genetic variability in isolates from 28 clinical cases (27 dogs and one cat) with otitis/dermatitis (157 different genotypes in 216 colonies; 3 to 12 colonies picked up per sample from each animal). All the animals had polyclonal otitis or dermatitis, with 3 to 10 different genotypes recovered per sample. In contrast, the MICs were much less variable (e.g., 0.031–0.063 µg/mL for ITZ for most colonies) and did not differ or differed in just one two-fold dilution for different genotypes from the same animal. A particular finding that reinforces the lack of correlation between genotype and antifungal susceptibility is the 12 colonies with the highest MICs of some agents (FCZ > 64 μg/mL; KTZ and PSZ 2–4 μg/mL) not sharing the same genetic profile but rather the same source. The colonies belonged to nine different genotypes and came from the same dog with chronic otitis.

## 9. MICs Higher for Isolates Developing as “Biofilm”

Biofilms are differentiated microorganism communities formed by a single microbial agent or by a mixture of fungal and bacterial species. Biofilms adhere to a biotic or abiotic surfaces, and its structure contributes to the innate physical and chemical resistance of the microorganisms [103]. Some studies [55,57,63,65] demonstrated that most isolates of *M. pachydermatis* can produce a biofilm and, as shown for other organisms [104], in this form the yeast has significantly reduced antifungal susceptibility—e.g., the MICs increase from 0.03 to >16 µg/mL for both KTZ and ITZ [57].

Some authors gave these results great clinical importance. Figueredo et al. (2013) [63] stated, “It is known that canine *Malassezia* infections are usually chronic, with conventional therapy largely ineffective. This may be due to the ability of *Malassezia* to form biofilms, consequently requiring higher drug concentrations than are currently used to cure infection.” Bumroongthai et al. (2016) [57] even emphasised the need to use, in addition to antifungal therapy, topical treatments capable of removing *Malassezia* biofilm from the skin of dogs. We think that more caution is necessary for drawing such conclusions. The claim that “conventional therapy” would be “largely ineffective” in the treatment of *Malassezia* infections in dogs lacks any support. Instead, veterinary dermatologists widely think that cases of MD and MO become “chronic” when primary causes and predisposing factors remain uncorrected [18,96]. It must be stressed that the yeasts (and bacterial infections) of the ear canals and the skin are thought to be secondary problems in most cases [6,19].

Most importantly, the biofilm was obtained only in vitro, in microplate wells [57,63,65] or on the surface of segments of catheters [65]. Additionally, in other past studies [105,106], the production was demonstrated only in vitro. As underlined by Pye et al. (2013) [104] in their study reporting biofilm producers among *Pseudomonas aeruginosa* isolates, while the microtitre plate assay is widely used, it is clearly an artificial system that cannot mimic the complexity of the microenvironment of an inflamed ear.

Based on the above considerations, it would be necessary to clarify whether and how often *M. pachydermatis* can produce a biofilm on the skin and in the ears of dogs. We believe that this possibility should be explored with regard to, in particular, the ear localisation. A quite common finding in the course of otitis, especially in chronic forms, is the presence in the ear canal of abundant material composed of exudates, cerumen, and debris in which bacteria and yeasts can be harboured. It is considered of fundamental importance to apply medicaments able to penetrate (or remove) this material to allow antimicrobials to reach effective local concentrations [18]. According to Nuttall [107], this material would represent, more often than you think, an actual biofilm. In particular, the biofilm would be easy to recognise clinically as an adherent, thick, and slimy discharge that is frequently dark brown or black, and on cytology as a variably thick veil-like material including bacteria and cells [107]. The observation of bacterial biofilms directly in different infection foci has also been reported in human medicine [108].

**Figure 5:**
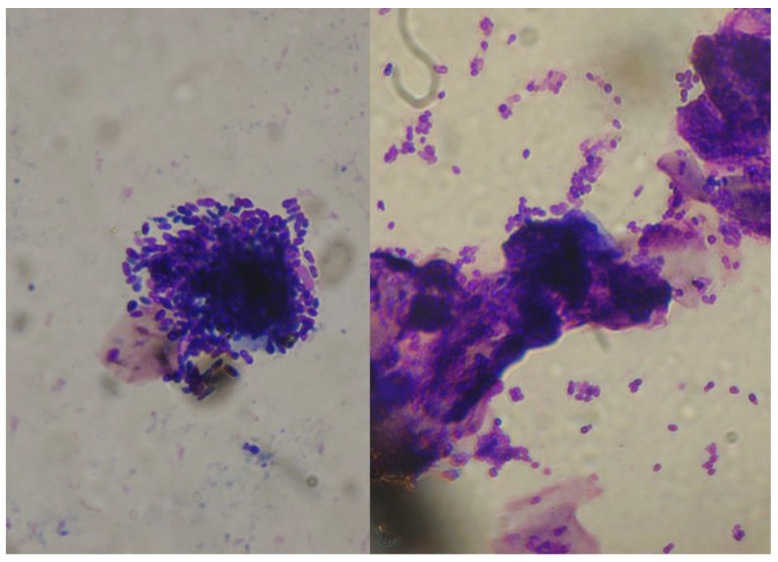
Cytology from ear discharge in two dogs with *Malassezia* otitis. Clumps including numerous yeast cells (400-fold magnification, Hemacolor^®^ rapid staining kit).

In the case of MO, it is common to detect, on cytology from ear discharge, clumps including numerous yeast cells and amorphous material (Figure 5). These findings may be due to the active overgrowth of the yeasts which remain “entrapped” in the cerumen and debris. However, it would be interesting to investigate the possibility that, at least in some cases, these clumps correspond to the sites of the formation of actual biofilm.

A final consideration is that the results regarding the biofilm of *M. pachydermatis* have high relevance if applied to a completely different clinical entity, namely bloodstream infections in human neonates. Episodes have increased in recent years [109], and the use of intravenous catheters has been recognised as a significant risk factor, as they may be colonised by yeast [110].

## 10. Induced “Resistance” (In Vitro)

Jesus et al. (2011) [51], using the technique described by Fekete-Forgács et al. (2000) [111] (which consists of exposing a microorganism to increasing concentrations of a drug in a broth medium) reported a dramatic increase in the MICs of FCZ, KTZ, and ITZ against 30 isolates of *M. pachydermatis* (e.g., ITZ MIC_90_ for original strains 0.5 µg/mL; after induction 64 µg/mL). Interestingly, they exposed the isolates only to FCZ, which exhibits the possibility of cross-resistance phenomena among various azoles.

Similar results were obtained in another study [49] using the same approach [111] to induce “resistance” in one isolate that was used to investigate the suitability of different growth media in a broth-dilution procedure. Nakano et al. (2005) [25] obtained increased MICs using a different approach. They performed 30 subcultures in the presence of drug concentrations (KTZ, NYS, TER) around the MIC. Other studies generated mutants resistant/tolerant to NYS [74], KTZ [72], and MCZ. [112] to investigate possible mechanisms of resistance (see Section 12) using the following methods: exposure to N-methyl-N’-nitrosoguanidine and UV radiation [74] and serial subcultures of a yeast colony on a solid medium containing increasing concentrations of KTZ [72] and MCZ [112].

Overall, these findings indicate that *M. pachydermatis* is capable of developing resistance mechanisms. However, to what extent the in vitro conditions employed to induce resistance mimic what occurs to *Malassezia* upon exposure to drugs during the treatment of a dog remains to be determined, though Jesus et al. (2011) [51] claimed that their approach simulated a “situation that may occur in veterinary clinic patients under azole therapy”. Regardless of these considerations, in the study by Nakano et al. [25] even before the in vitro induction of resistance, the MICs of TER reached very high values (range 0.39–25 µg/mL), indicative of potential clinical resistance in the case of oral administration (especially taking into account the low levels reached by TER in the stratum corneum and sebum of dogs [97]).

## 11. Proposal for Tentative ECVs

As mentioned above, ECVs represent the upper limit of the WT MIC distribution. They are intended as cut-offs to track the emergence of non-WT isolates, namely those that may harbour some resistance mechanisms [79]. A recent study [60] proposed tentative ECVs for *M. pachydermatis* (32, 0.032, and 0.064 µg/mL for FCZ, ITZ, and PSZ, respectively) that were calculated using 62 isolates and according to the rule that the ECV is generally two doubling dilutions above the modal value in a MIC distribution [52,79]. The authors of another study [55] employed these ECVs and reported 10 (40%) and 11 (44%) isolates out of 25 as potentially non-WT in regard to KTZ and ITZ, respectively.

This way of looking for interpretive criteria as alternatives to CBPs has a sound rationale. For example, for *Candida* ECVs have been proposed as a means to track isolates potentially resistant to antifungal agents, such as AMB, for which CBPs are not available [52]. However, these tentative ECVs for *Malassezia* [60] may not be very informative on a global scale because they were calculated based on MIC values that may not be reproducible with another testing method. Moreover, the results of a single laboratory were included, though there is a consensus in the international literature to determine ECVs based on data from at least 3 to 5 different laboratories to avoid biases related to the use of isolated data and taking into account possible inter-laboratory differences [79]. The value of ECVs for *Candida* lies in their establishment from the analysis of global antifungal surveillance databases that include hundreds or thousands of MICs obtained in different laboratories [52,79].

## 12. Possible Mechanisms of Resistance in *M. pachydermatis*

Uchida et al. (1994) [74] showed that the quantity of membrane sterols in NYS-resistant mutants of the reference strain CBS 1879 (resistance induced in vitro) was significantly decreased compared to the original strain. As sterols are the main target of polyene antifungal agents—which include NYS [12]—the authors hypothesised that this reduction directly correlated with the increased MICs observed for the resistant mutants. Interestingly, the proportion of fecosterol in mutants was significantly increased, which may suggest a mechanism of resistance similar to the one described for *Candida* against azoles (the development of bypass pathways, through which ergosterol is replaced by its precursor 14a-methylfecosterol, with the latter product leading to still-functional membranes [39]).

Another study [26] reported that the defence mechanisms against azoles by *M. pachydermatis* might depend on efflux pumps—a common mechanism of azole resistance in *Candida* species [113]—particularly those belonging to the “major facilitator superfamily”. This was shown by the combination of FCZ and a substance able to inhibit these pumps (haloperidol (HAL)), resulting in an increased in vitro drug activity (MIC for 14 isolates: without HAL 8–512 µg/mL; with HAL 2–64 µg/mL).

Kano et al. (2019) [83] showed that an isolate with proved clinical resistance (see Section 4) had missense mutations in the ERG11 gene that encodes lanosterol 14 α demethylase, the target site for antifungal azoles. Mutations in the same gene were described in two field isolates with multi-azole in vitro resistance [85] and MCZ-resistant clones of CBS 1879 selected by serial passages on MCZ-supplemented media [112].

Another recently proposed possibility is that of chromosomal rearrangement and gene over-expression, which are quite common mechanisms of resistance in other fungal species [114]. Kim et al. (2018) [72] found that a region in chromosome 4 of two isolates of *M. pachydermatis*—a field isolate with a high MIC of KTZ and an in vitro generated mutant—was tandemly quadruplicated. This rearrangement resulted in an increased expression of the genes contained in the region, including *ERG11* and *ERG4,* which are involved in the pathway targeted by KTZ.

A final consideration regards the possibility that a lower azole susceptibility occurs due to the uptake of exogenous sterols provided by sebum or cerumen. The ability to import and use host sterol when ergosterol biosynthesis is blocked is considered a likely cause of the azole resistance typical of certain yeast species, such as *Candida glabrata* [115].

This mechanism of resistance may be present in the Malassezia species, too, as the members of this species cannot produce fatty acids themselves but need lipids from the environment for growth [34]. As regards specifically *M. pachydermatis*, evidence in support of this possibility may come from the results of some in vitro studies. For example, Jesus et al. [51] reported good growth in RPMI broth, which is surprising since this medium is lipid-free. According to the authors [51], the subcultures that they performed on a lipid enriched medium (Dixon agar) before testing avoided the depletion of the lipid reserves of the yeast and allowed the subsequent growth in RPMI.

This information enables also pointing out a technical issue. The complex lipid-supplemented media employed for the susceptibility testing of *M. pachydermatis* (or for subcultures of isolates before the execution of the tests) may allow the fungal strains to accumulate sterols as reserves in lipid droplets [116]. These reserves may, in turn, affect the results of antifungal tests for drugs acting on ergosterol yeast levels. 

## 13. Conclusions and Perspectives

The key points of this review are summarised in Table 3. Though the results of several studies support the capacity of developing resistance mechanisms, it is difficult to estimate how much importance should be given now to the problem of “antifungal resistance” in *M. pachydermatis*. This point will be clarified only when a reference AFST method and clinical breakpoints are available. The experience gained from other organisms indicates the necessity of a multicentre approach, maybe under the auspices of a leading organisation, such as the International Society for Human and Animal Mycology (ISHAM) or the Veterinary Committee on Antimicrobial Susceptibility Testing (VetCAST). With a shared strategy, it should be possible to obtain a test able to provide intra- and inter-laboratory reproducible MIC data to guide therapeutic decision-making and epidemiological analyses.

## Figures and Tables

**Table 1:** Antifungal agents used to treat *Malassezia* dermatitis and otitis in the dog.

Mechanism of Action	Class	Agent	Use *	Notes
Interaction with sterol-14α-demethylase, involved in the biosynthesis of ergosterol	Azole derivatives (imidazoles)	KTZ	O/T	Recognised clinical efficacy for canine *Malassezia* dermatitis (oral administration).
		MCZ	T	Principally available in aural formulations. Available in formulations for dermatological use in many countries.
		CTZ	T	Principally available in aural formulations.
		ECZ	T	-
	Azole derivatives (triazoles)	ITZ	O	Recognised clinical efficacy for canine *Malassezia* dermatitis (oral administration).
		FCZ	O	Traditionally employed in the case of systemic mycoses. One study has demonstrated some efficacy in treating *Malassezia* dermatitis.
		PSZ	T	Used mainly in human medicine for the treatment of invasive fungal infections. An aural PSZ-based formulation for dogs has also been recently marketed.
Action against membrane sterols	Polyene macrolides	NYS	T	Principally available in aural formulations.
Perturbation of fungal sterol synthesis by inhibiting squalene epoxidase	Allylamines	TER	O/T	Found effective for *Malassezia* dermatitis with oral treatment in some studies. Available for topical use in some countries.
Inhibition of microtubule assembly	Benzimidazoles	TBZ	T	Principally available in aural formulations.

KTZ = ketoconazole; MCZ = miconazole; CTZ = clotrimazole; ECZ = econazole; ITZ = itraconazole; FCZ = fluconazole; PSZ = posaconazole; NYS = nystatin; TER = terbinafine; TBZ = thiabendazole. * The availability of formulations containing the different agents varies according to country. (O = oral; T = Topical) Data from: [4,9,10,11,12,13,14,15,16,17,18,32].

**Table 2:** Summary information regarding 7 cases of treatment failure due to antifungal resistance of *M. pachydermatis* (see Document S1 for further details).

Animals and Country	In Vitro Methods	Results	Ref.
Five dogs with *Malassezia* otitis/dermatitis. Resistance suspected because of failure to respond to typically clinically effective topical antifungal therapies (CTZ [2 cases], MCZ [2 cases], CTZ, and KTZ [1 case]). Sample sites: ear (for one dog, also an isolate obtained from the interdigital area was employed) (Australia)	Broth micro-dilution method using two procedures and a disk diffusion method. Antifungal agents tested: CTZ, MCZ, NYS.	A correlation of clinical lack of response with in vitro results was noted. The MICs of antifungal agents for which resistance was perceived were > 32 µg/mL (i.e., a MIC was not obtained even at the highest concentration tested). These results were confirmed by the disk diffusion method for MCZ (all isolates were considered R according to the guidelines of the producer of the disks, with most strains producing no zone of inhibition). Less consistent results were obtained for CTZ. MICs of NYS (range 2–16 µg/mL) were instead obtained for all isolates, which were considered susceptible to NYS also according to the results of the disk diffusion method. Two control isolates, where empirically selected antifungals were clinically effective, did not show evidence of resistance on in vitro tests.	[84]
15-year-old neutered Miniature Dachshund. Resistance suspected because of the lack of response despite oral therapy with ITZ and topical treatment with MCZ (shampoo) (Japan).	Broth micro-dilution method and the commercial method E-test^®^. Antifungal agents tested: ITZ, KTZ, MCZ, CTZ.	MICs of ITZ, KTZ, MCZ, CTZ (>32 µg/mL) were increased by several-fold compared with MICs obtained for the control isolates. The presence of resistance phenomena was supported by the finding of missense mutations in the gene ERG11 encoding the drug target enzyme (sterol 14*α*-demethylase).	[83,85]
5-year-old neutered female toy Poodle treated continuously for 2,5 years with systemic (ITZ) and topical (MCZ shampoo and ear drops) azoles to control an “idiopathic” form of *Malassezia* dermatitis/otitis. Resistance suspected because of the loss of treatment efficacy. Isolates tested in vitro were obtained from 3 body sites (Italy).	Broth micro-dilution method and the commercial method E-test^®^. Antifungal agents tested: CTZ, MCZ, ITZ, KTZ, FCZ, PSZ.	The MICs of different azoles—in particular, ITZ, KTZ, and MCZ—were increased by several-fold compared with the MICs obtained for the control isolates (i.e., isolates of the yeast coming from dogs never subjected to antifungal therapies and a reference strain). No activity of MCZ and ITZ (MIC> 32 µg/mL) was found for one of the isolates.	[82]

CTZ = clotrimazole; FCZ = fluconazole; KTZ = ketoconazole; ITZ = itraconazole; MCZ = miconazole; PSZ = posaconazole.

**Table 3:** Key points of the review.

It is proved that *M. pachydermatis* can develop resistance phenomena leading to treatment failure in a dog with *Malassezia* otitis and dermatitis.
The antifungal agents more commonly involved in phenomena of resistance are azole derivatives.
In the few well-documented cases, the treatment failure occurred after months or years of therapy, which may indicate that resistance in *M. pachydermatis* is an acquired slow-developing phenomenon.
Cross-resistance of *M. pachydermatis* to different azoles—a phenomenon demonstrated during in vitro experiments—may also occur in vivo.
Further evidence in support of the capacity by *M. pachydermatis* of developing resistance comes from: ✓ The higher MICs found for isolates from animals with probable/confirmed exposure to antifungal drugs and isolates exposed to antifungal agents in vitro; ✓ The description of possible resistance mechanisms in field isolates and in mutant isolates obtained in vitro; ✓ The reports of isolates with MICs significantly higher (or no MIC at all) within a certain population of isolates.
Most strains of *M. pachydermatis* can produce biofilm in vitro, and in this form the yeast has a significantly reduced antifungal susceptibility. The clinical relevance of this finding remains to be determined.
It is difficult to estimate how much importance should be given now to the problem of “antifungal resistance” regarding *M. pachydermatis*: ✓ The data reviewed may suggest than the development of resistance is a rare eventuality in dogs with Malassezia otitis and dermatitis. For example, only three publications describe confirmed cases of treatment failure due to antifungal resistance, and most claims of resistance made by past studies are based on interpretive breakpoints that lack sound support in clinical perspective. ✓ However, some considerations highlight the need for surveillance and vigilance for the possible emergence of clinically-relevant resistance: ▪ Resistant cases may be more widespread than you think and simply underreported in official literature (this may be due, for example, to the difficulty in obtaining a laboratory confirmation in the absence of standardised susceptibility methods); ▪ *Malassezia* otitis and dermatitis often necessitate frequent and lengthy treatment courses (especially in the cases of atopic dermatitis, seborrhoeic dermatitis, and chronic otitis externa). The chronicity of treatment provides ideal opportunities for the selection of resistance; ▪ Though we attempted to identify possible contributions from “grey literature” from all over the world (by sending a request to an international dermatological forum), we may have overlooked some publications regarding cases of resistance (e.g., publications in non-Western countries).

## Supplementary Materials

The following are available online at https://www.mdpi.com/2309-608X/6/2/93/s1, Document S1: Description of the studies included in the review.
